# Hopelessness as a Mediator of the Association Between Parenting Factors and Adolescent Suicidality and Substance Use Among Juvenile Justice‐Referred Youth

**DOI:** 10.1002/mhs2.70000

**Published:** 2025-02-11

**Authors:** Natalie Guerrero, Lauren O'Reilly, Trey V. Dellucci, Casey Pederson, Zachary W. Adams, Leslie Hulvershorn, Tamika C. B. Zapolski, Matthew C. Aalsma

**Affiliations:** ^1^ Department of Pediatrics, School of Medicine Indiana University Indianapolis Indiana USA; ^2^ Department of Psychiatry, School of Medicine Indiana University Indianapolis Indiana USA; ^3^ Adolescent Behavioral Health Research Program, School of Medicine Indiana University Indianapolis Indiana USA

**Keywords:** hope, juvenile justice‐referred youth, parental monitoring, parental support, substance use, suicidality

## Abstract

Youth involved in the juvenile justice system are more likely to have a substance use disorder and/or suicidality (e.g., suicidal thoughts and behavior) compared to other youth. Although parental support and monitoring may play an important role in youth substance use and suicidality outcomes, the potential mechanisms have not been elucidated. Our purpose was to evaluate the extent to which parental support and monitoring were associated with latent, continuous construct scores of suicidality and substance use and to determine whether youths’ hopelessness may indirectly affect these relationships among a sample of youth referred to the juvenile justice system. The sample included juvenile justice‐referred youth aged 14–17 (*N* = 77; 69% White, 58% male, 74% non‐Hispanic). The primary predictors of interest were parental support and monitoring, measured by the Parent Support Scale and Parental Monitoring Scale. The primary potential mediator of interest was hopelessness. Linear regression was used to model continuous suicidality and substance use severity scores, measured via a computer adaptive test, on parental support and monitoring. We tested hopelessness as a potential mediator. All analyses controlled for age, sex assigned at birth, race, ethnicity, and family income. After adjustment, parental support was associated with decreased suicidality severity (*β* = −0.30, *p* = 0.002). Parent support and monitoring were associated with youth‐reported hopelessness. The indirect mediation effects of hopelessness in the relationship between parental support (*β* = −0.18 [SE, 1.73]), as well as parental monitoring (*β* = −0.17 [SE, 0.20]), and suicidality severity were statistically significant. Parental support and youth hopelessness may be important intervention targets for improving and addressing disparities in substance use and suicidality among juvenile justice‐referred youth. Hope‐based interventions may be effectively integrated into existing juvenile justice programs, and their potential to improve both mental health and behavioral outcomes among justice‐involved youth should be examined.

## 1 Introduction

### 1.1 Substance Use and Suicidality Among Youth

Drug overdose and suicide are the leading causes of death among youth in the United States (Gitterman, Hay, and Langford 2023). This is consistent with population health trends, as the rate of drug overdose deaths and poisoning among children and youth in the United States increased significantly in recent years (Goldstick, Cunningham, and Carter 2022), and suicide rates among youth continue to be high (Centers for Disease Control and Prevention, National Center for Health Statistics 2024; Centers for Disease Control and Prevention 2024; Panchal 2024). Youth with legal involvement experience disproportionately high rates of both substance use and suicidality compared to other youth, presenting serious public health concerns. Approximately 25% of adolescents aged 12–17 years who have been in jail or detention centers have used alcohol, tobacco, or other substances in the past year (RTI International 2003) and nearly half of first‐time offending, court‐involved, nonincarcerated youth endorse active substance use (Tolou‐Shams et al. 2019). Furthermore, rates of past‐year suicidal ideation (estimates range from 19% to 32%) and suicide attempts (estimates range from 12% to 15.5%) are greater among youth involved in the juvenile justice system compared to other youth (Stokes et al. 2015; Scott, Underwood, and Lamis 2015).

The disproportionate prevalence of both substance use and suicidality among legally involved youth can be understood within the syndemics framework, which describes how two or more disorders can co‐occur, interact, and amplify disease burden within a specific population (Singer 2009) because of familial, social, and political factors that contribute to a negative environment which creates further vulnerabilities for disease among minoritized groups (Mendenhall 2017). Consistent with this, youth involved in the justice system experience overlapping risk factors, such as exposure to trauma, family instability, and social disadvantage. Additionally, the presence of both mental health challenges and substance use in these youths can compound difficulties, intensifying the risk of severe outcomes. Substance use and suicidality share behavioral mechanisms, such as impulsivity and poor coping skills, which can heighten vulnerability in these youth. Given these interconnected factors, investigating both issues together may provide a clearer understanding of intervention needs and effective strategies for this population. Although youth involved in the juvenile justice system have an increased likelihood of having a substance use disorder and suicidal thoughts compared to other youth, there is relatively limited research focused on youth involved in the juvenile justice system compared to general community samples of youth.

### 1.2 Parental Monitoring: Associations With Substance Use and Suicidality

Parental monitoring is a modifiable behavior in intervention for both substance use and suicidality (MacPherson et al. 2021; Pelham et al. 2024; Keogh‐Clark et al. 2021; McGillicuddy and Eliseo‐Arras 2012) and has been described as a caregiver's attention and tracking of their child's behavior (Dishion and McMahon 1998). It has been linked to lower rates of substance use in general adolescent samples. It may be an important protective factor for substance use, as monitoring may help to discourage teens from using a variety of substances and allow caregivers to notice and intervene early in the development of adolescents’ substance use behavior (Pelham et al. 2024; Keogh‐Clark et al. 2021). By comparison, decreased parental monitoring has been associated with greater substance use initiation among youth who initially denied using substances (Pelham et al. 2024). Parental monitoring also has potentially positive impacts on suicide‐related outcomes. A meta‐analysis of 31 studies found that such relationships are associated with lower levels of suicidal ideation among youth (Yang et al. 2023). Parental monitoring may also provide a foundation for improved outcomes in the setting of mental health treatment. For example, a randomized controlled trial of integrated cognitive‐behavioral therapy compared to standard treatment included adolescents with comorbid substance use disorders and psychiatric disorders. It found that higher levels of parental monitoring at baseline predicted an improved trajectory of suicidal ideation (MacPherson et al. 2021). Additionally, parental monitoring has been inversely associated with suicidal attempts among a population of detained youth (Rohde, Seeley, and Mace 1997).

Furthermore, parental monitoring may similarly impact substance use outcomes among youth involved in the legal system, although studied less extensively. Early studies examining the role of parental monitoring on substance use among justice‐involved youth specifically have also found that increases in parental monitoring were associated with reductions in substance use, including cannabis and opioid misuse (Voisin et al. 2012; Vroom and Johnson 2024; Micalizzi et al. 2022), indicating a protective role against substance‐related harm. A similar pattern has been observed between parental support and suicidality among youth involved in the legal system. Studies have found that minimal parental support was associated with increased suicidal ideation and behavior (Stokes et al. 2015). Together, these studies suggest that parental support may be a protective factor against substance use and suicidality among these youth.

### 1.3 Parental Support: Associations With Substance Use and Suicidality

Parental support is another parental behavior that is associated with decreased adolescent substance use (Wills and Yaeger 2003) but has been overlooked in the literature on youth involved in the legal system. Parental support represents a caregiver's ability to promote behaviors through encouragement, co‐activity, and logistic planning (Rhodes et al. 2015). Family dynamics may be particularly impactful for these youth, who face unique stressors related to their justice involvement. A supportive family environment could counterbalance the negative effects associated with legal involvement, offering a buffer against escalating risk behaviors. Although the literature on parental support among legal‐involved youth is small, there is evidence that youth substance use varies across dimensions of parental warmth and hostility (Bosk et al. 2021), which may be a proxy for parental support. While parental warmth was associated with lower use of illicit drugs, parental hostility was associated with greater use (Bosk et al. 2021; Robillard et al. 2022). Another study of legal‐involved youth examined a global scale of family functioning—which was inclusive of parental support—and found that higher levels of family functioning were associated with lower alcohol and cannabis use (Folk et al. 2020).

### 1.4 Potential Mechanisms of Action

The path by which parental support and monitoring impact substance use and suicidality among youth involved in the legal system is also not well understood. Therefore, this study aims to examine the extent to which parental support and monitoring influence suicidality and substance use severity in these youth, with hopelessness as a potential mediator. Hope is the cognitive belief that one can achieve their desired goals (Snyder 2000), and hopelessness may be a mechanism in the development of suicidal ideation as well as substance use. Adolescent hopelessness is influenced by parenting behaviors including parental support (Mahon and Yarcheski 2017) and has been identified as a critical predictor of both suicide and substance use (Pompili et al. 2012). However, the role of hopelessness as a mediator in this context is yet to be explored in detail. By focusing on hope as an intermediary factor, this study seeks to uncover how family‐based protective factors might be leveraged to improve mental health outcomes for youth involved in the legal system. Based on previous research, we hypothesize that hopelessness may indirectly explain the associations among parenting factors and youth substance use and suicidality.

## 2 Methods

### 2.1 Data Source

Data were drawn from the baseline assessment of an implementation study, the Family Based Justice Improvement Project (FB‐JIP), which aimed to increase universal substance use screening for juvenile justice‐referred youth on probation (Aalsma et al. 2019). Participants included juvenile justice‐referred youth and their caregivers. Participants were recruited from two partnered counties after youth were arrested and referred to the justice system. They were recruited at intake, first being provided a flyer with study information and then contacted by study personnel in the days after returning home. To be eligible for the study, youth needed to be between 14 and 17 years old and involved in the justice system in one of two Midwest counties who were released to the community under formal probation supervision, under informal adjustment, or under no supervision. Youth also needed to report a lifetime history of substance use and score a one or higher on the CRAFFT, a clinical screening tool for identifying substance use disorder risk among children and adolescents (Knight et al. 2002). Youth were excluded from the study if they were currently detained, already enrolled in outpatient substance use treatment, and if the youth or caregiver was unable to communicate in English. Eligible youth provided their assent, and their caregivers provided consent to participate in the study. Participating youth were compensated $25 and caregivers $25 for completing the baseline assessment. The Indiana University Institutional Review Board approved the study.

### 2.2 Measures

The primary outcome of interest was suicidality and substance use, as measured by the Kiddie‐Computerized Adaptive Test (K‐CAT), a computer‐adaptive test that comprehensively screens for a variety of mental health and substance use symptoms and behaviors and is validated for youth across populations (Gibbons, Kupfer et al. 2020; Cervantes 2024; Gibbons, Alegria et al. 2020; Adams et al. 2024). Its approach uses multi‐dimensional item response theory (MIRT) to efficiently identify suitable item subsets for each respondent, where more severe items are weighed more heavily than less severe items. We used a continuous measure of severity for both suicidality and substance use; estimated severity is reported on a 0–100 scale, with increasing scores indicating greater severity. With regards to the suicidality scale, adaptive tests were drawn from a bank of 65 nonsuicide items and 10 suicidality items. Example items from the suicidality scale include “In the past two weeks, I felt everyone would be better off without me” and “In the past two weeks, I thought about killing myself.” For substance use, adaptive test items were drawn from a bank of 168 items that allowed for the calculation of an overall severity score, rather than a severity score for each substance endorsed. Youth were asked whether they had used (yes/no) the following substances: alcohol, cannabis, opioids, cocaine, methamphetamine/amphetamine, sedatives, hallucinogens, and nicotine. If the youth responded “yes,” the youth were then asked questions about that specific substance. Sample items include, “When I stop using cannabis/marijuana/‘spice’ I get withdrawal symptoms (e.g., grouchy or down mood, anxiety, sleep problems, restlessness, shakiness, feeling sick).”

The primary exposures of interest were parental support and parental monitoring as reported by adolescent participants. Parent support was measured by the Parent Support Scale (PSS) (Lonardo et al. 2009). In the youth survey, there were 10 items, such as “My parent often asks me what I am doing in school,” “My parent trusts me,” as well as items that were reverse scored, such as “My parent sometimes puts me down in front of other people,” and “My parent is clueless about a lot of things I do.” Cronbach's *α* for parental support measures was 0.87. Parental monitoring was measured by the Parental Monitoring Scale (PMS) (Huebner and Howell 2003). PMS items included eight statements, such as “My parent knows where I am after school,” “I tell my parent whom I'm going to be with before I go,” and “My parent knows who my friends are.” Two additional items not part of the original PMS were included: “My parent monitors my cell phone use,” and “My parent looks at my social media profiles.” A mean score was determined, with higher scores indicating greater monitoring. Cronbach's *α* for parental monitoring measures was 0.82.

The primary potential mediator of interest was hopelessness as reported by the youth. The hopelessness measure was derived from the Flourishing Children Positive Indicators Project developed by the nonprofit research organization, Child Trends (https://childtrends.org/) (Lippman 2014). Hopelessness was measured by three items: “I expect good things to happen to me,” “I am excited about my future,” and “I trust my future will turn out well.” A mean score was determined, with higher scores indicating greater hopelessness. Cronbach's *α* for hopelessness measures was 0.87 (Child Trends Hope 2024).

Other measured variables included age (age 14 [reference], 15, 16, 17), sex assigned at birth (female [reference], male), race (White [reference], Black/African American, American Indiana/Alaskan Native, Asian, Other), and ethnicity (non‐Hispanic/Latinx [reference], Hispanic/Latinx). Individuals could endorse more than one race. For analyses, race was dichotomized into White or any minority racial identification. Family household income was measured by the Family Affluence Scale, which is a validated, four‐item, adolescent, self‐report measure of family wealth (Boyce et al. 2006). Scores are totaled and range from 1 to 9.

### 2.3 Analysis

**Figure 1:**
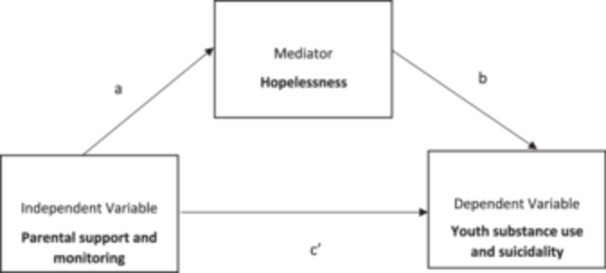
Hopelessness as a potential mediation of the relationships between parenting factors and youth substance use and suicidality.

All analyses were conducted in R version 4.3.1. We first conducted multiple imputations using the mice package (version 3.16.0) specifying 25 imputations and predictive means matching. We then conducted mediational analyses using structural equation modeling with the lavaan package (version 0.6‐17). We used linear regression to model suicidality and substance use on parental support and parental monitoring. In Figure 1, we show that we included hopelessness as a potential mediator using the methods proposed by Zhao, Lynch, and Chen (2010). We included potential confounding variables in the models to better isolate these effects (age, sex assigned at birth, race, ethnicity, and family income). In the mediational analyses, two additional parameters were defined: (1) an indirect effect, calculated as the product between the predictor and mediator (a) and mediator and outcome (b) paths, and (2) the total effect, calculated as the sum of the direct path between the predictor and outcome (c) and the indirect path (a × b). Standard errors were bootstrapped using 500 samples. Standardized estimates were reported. The study was not preregistered. Analytic code can be accessed in a GitHub repository (https://github.com/LaurenMOReilly/FBJIP_SUD_SUI). Due to the legal involvement of the youth included in the study, the data set is not made publicly available.

## 3 Results

Table 1 provides the demographic information of the youth sample (*N* = 77), as well as imputed results to account for missing data. Most were male (58.4%) and identified as non‐Hispanic (84.4%) and White (68.9%), although a large portion identified as Black/African‐American (26%).

**Table 1:** Demographic information of sample.

	Non‐imputed dataset	Imputed dataset
	*N*, %^a^	*N*, %^a^
Age		
14	15 (19.5)	15 (19.5)
15	18 (23.4)	18 (23.4)
16	23 (29.9)	23 (29.9)
17	21 (27.3)	21 (27.3)
Gender		
Female	31 (40.3)	32 (41.6)
Male	45 (58.4)	45 (58.4)
Missing	1 (1.3)	—
Race^b^		
White	53 (68.9)	53 (68.9)
Black/African American	20 (26.0)	20 (26.0)
American Indian/Alaskan Native	3 (3.9)	3 (3.9)
Asian	1 (1.3)	1 (1.3)
Other	10 (13.0)	10 (13.0)
Ethnicity		
Non‐Hispanic/Latinx	57 (74.0)	65 (84.4)
Hispanic/Latinx	9 (11.7)	12 (15.6)
Missing	11 (14.3)	—
	M (SD)	M (SD)
Family household income	4.2 (1.6)^c^	4.2 (1.6)
Parent support scale	2.4 (0.7)^a^	2.4 (0.7)
Parental monitoring scale	36.9 (7.3)^a^	36.9 (7.3)
Hopefulness scale	8.0 (3.5)^a^	8.0 (3.5)
K‐CAT suicidality	35.2 (22.5)^d^	35.4 (22.3)
K‐CAT substance use	38.1 (18.3)^e^	38.6 (19.0)

^a^ Based on 77 unique individuals.

^b^ Note that individuals could endorse more than one race; percentages ≥ 100%.

^c^ Based on 76 unique individuals.

^d^ Based on 75 unique individuals.

^e^ Based on 67 unique individuals.

### 3.1 Parental Support

Table 2 provides the results of the structural equation models for the role of parental support on mental health outcomes. When examining the direct path between predictor and outcome, parental support was statistically significantly associated with suicidality severity (*β* = −0.19 [SE, 0.09]), but not substance use severity. Parental support was negatively associated with hopelessness, both for suicidality and substance use (*β* = 0.41 [SE, 0.11]). The indirect effect of hopelessness in the relationship between parental support and suicidality was statistically significant (*β* = −0.18 [SE, 0.06]). The total effect among parental support, hopelessness, and suicidality (*β* = −0.37 [SE, 0.09]) was statistically significant. Neither the indirect nor total effect was statistically significant when examining substance use.

**Table 2:** Mediation analyses between parental support and adverse outcomes.

	Path	Parameter estimate (SE)	*p* value
Direct effects			
Model 2a: parental support, hopelessness, suicidality			
Parental support scale (PSS) → hopelessness scale	a	−0.41 (0.11)	0.000
Hopelessness scale → K‐CAT suicidality	b	0.44 (0.09)	0.000
Parental support scale → K‐CAT suicidality	c′	−0.19 (0.09)	0.041
Indirect effect	a × b	−0.18 (0.06)	0.001
Total effect	c′ + (a × b)	−0.37 (0.09)	0.000
Model 2b: parental support, hopelessness, substance use			
Parental support scale → hopelessness scale	a	−0.41 (0.11)	0.000
Hopelessness scale → K‐CAT substance use	b	0.17 (0.13)	0.164
Parental support scale → K‐CAT substance use	c′	0.08 (0.11)	0.471
Indirect effect	a × b	−0.07 (0.06)	0.209
Total effect	c′ + (a × b)	0.01 (0.12)	0.937

*Note: N* = 77. PSS = mean score. K‐CAT is continuous score. Covariates included age, gender, race, ethnicity, and household income. Standard errors are bootstrapped using 500 samples. Standardized results presented.

### 3.2 Parental Monitoring

Table 3 provides the results of the structural equation models for the role of parental monitoring on mental health outcomes. Direct paths between parental monitoring and suicidality or substance use were not statistically significant. Parental monitoring was negatively associated with hopelessness in suicidality and substance use models (*β* = 0.33 [SE, 0.10]). Hopelessness was negatively associated with suicidality (*β* = −0.51 [SE, 0.09]). The indirect effects of hopelessness in the relationship between parental monitoring and suicidality (*β* = −0.17 [SE, 0.06]) were statistically significant. Similar to parental support, substance use did not demonstrate statistically significant direct or total effects.

**Table 3:** Mediation analyses between parental monitoring and adverse outcomes.

	Path	Parameter estimate (SE)	*p* value
Direct effects			
Model 3a: parental monitoring, hopelessness, suicidality			
Parental monitoring scale (PSS) → hopelessness scale	a	−0.33 (0.10)	0.000
Hopelessness scale → K‐CAT suicidality	b	0.51 (0.08)	0.000
Parental monitoring scale → K‐CAT suicidality	c′	−0.03 (0.10)	0.734
Indirect effect	a × b	−0.17 (0.06)	0.004
Total effect	c′ + (a × b)	−0.20 (0.11)	0.057
Model 3b: parental monitoring, hopelessness, substance use			
Parental monitoring scale → hopelessness scale	a	−0.33 (0.10)	0.001
Hopelessness scale → K‐CAT substance use	b	0.18 (0.13)	0.163
Parental monitoring scale → K‐CAT substance use	c′	0.11 (0.11)	0.304
Indirect effect	a × b	−0.06 (0.05)	0.199
Total effect	c′ + (a × b)	0.05 (0.11)	0.633

*Note: N* = 77. PSS = mean score. K‐CAT is continuous score. Covariates included age, gender, race, ethnicity, and household income. Standard errors are bootstrapped using 500 samples. Standardized results presented.

## 4 Discussion

These results underscore the importance of parental factors on substance use and suicidality among juvenile justice‐referred youth. Findings revealed that both parental support and parental monitoring were positively associated with hopelessness, which in turn were associated with lower suicidality severity. They also showed that, surprisingly, neither parental support nor parental monitoring was associated with substance use severity.

### 4.1 Substance Use: Associations With Parental Support and Monitoring

Although parental monitoring is thought to be one of the strongest predictors of substance use among youth generally (Pelham et al. 2024), the nonsignificant association between parental behaviors and substance use severity may reflect unique challenges faced by families with teens involved in the legal system. Unlike earlier studies that examined the occurrence of substance use (Voisin et al. 2012; Vroom and Johnson 2024), the current study examined parental behaviors as a predictor of substance use severity among juvenile justice‐referred youth who currently use substances. This specific methodological detail might have increased the likelihood of a selection bias that ultimately impacted our ability to detect a significant relationship. Furthermore, legal involvement can disrupt family dynamics and strain relationships, potentially diminishing the protective effects of monitoring and support in cases of ongoing substance use. Alternatively, it may be that parental monitoring alone is insufficient to address substance use severity in youth who have already begun using substances. This finding underscores the importance of exploring multifaceted approaches, such as combining family‐based interventions with individual counseling or substance use treatment, to address the complex needs of youth involved in the legal system. In the remainder of this discussion, we focus on the implications of our significant findings as they relate to suicidality.

### 4.2 Parental Support: A Predictor of Suicidality

Parental support, as measured in this study, likely reflects the youth's perception of closeness and trust with their parents, which is central to theories addressing suicide prevention. Our measure of parent support may relate to parental–youth connectedness, as the scale inquired about parental attention, affection, trust, and perception of closeness. Connectedness is also a related feature of psychological theories of suicide. For instance, in the Three‐Step Theory of Suicide, a sense of connectedness may offset feelings of isolation or burdensomeness that contribute to suicidal thoughts. The Psychological Theory of Suicide proposes that when there is a perceived lack of social connectedness (i.e., thwarted belongingness) in combination with a sense of burdensomeness, suicidal desire develops. Therefore, parental support could be a foundational element in fostering hopefulness, as it reinforces a youth's sense of belonging and trust in their caregivers (Van Orden et al. 2010; David Klonsky and May 2015; O'Connor and Kirtley 2018).

### 4.3 Parental Monitoring: A Predictor of Suicidality

In this study, higher levels of parental monitoring were associated with decreased hopelessness, which in turn was linked to lower suicidality. Our results suggest that youths' perception of parental monitoring may be related to hopelessness, which may be, in turn, related to suicidality (MacPherson et al. 2021). The relationship between parental monitoring and hopelessness aligns with findings that consistent involvement and interest from parents can enhance resilience in youth. Parental monitoring not only helps parents detect and respond to early signs of suicidality but may also contribute to fostering a positive mental outlook in youth. Greater parental monitoring may suggest greater parental involvement in their youths' lives (Moon, Kim, and Parrish 2020), greater awareness of youths' worsening mental health and suicidality symptoms, and greater proactiveness to intervene for care. The proactive involvement inherent in monitoring may help youth feel supported, contributing to their sense of safety and optimism about the future (Pelham et al. 2024; Voisin et al. 2012; Vroom and Johnson 2024).

### 4.4 Hopelessness: A Partial Mediator

The results supported that hopelessness may partially explain the association between parenting factors and suicidality severity among a juvenile justice‐referred youth sample. Based on the recommendations laid forth by Zhao, Lynch, and Chen (2010) to interpret mediation results, hopelessness appeared to support *complementary mediation* between parental support and suicidality, whereas hopelessness supported *indirect mediation* between parental monitoring and suicidality (Zhao, Lynch, and Chen 2010). These results emphasize the nuanced role of hopelessness and suggest it may act as a mediator, particularly in the relationship between parental behaviors and suicidality.

The findings align with prominent psychological theories of suicide, which conceptualize hopelessness as a key factor in developing suicidal ideation. The potential support for mediation among parental factors and suicidality aligns with prominent psychological theories of suicide. Both the Interpersonal Theory of Suicide (Van Orden et al. 2010) and Three‐Step Theory of Suicide (David Klonsky and May 2015) conceptualize hopelessness as a causal mechanism (in the presence of other psychological factors) in the development of suicidal ideation. Additionally, the Integrated‐Motivational‐Volitional Model (O'Connor and Kirtley 2018) of suicide proposes that hopelessness about the future may serve as a catalyst between feeling trapped and suicidal ideation. These theories propose that an individual's sense of connection and future‐oriented optimism can counteract the risk of suicide by providing protective mental frameworks.

The complementary mediation observed in our study between parental support and suicidality via hopelessness further suggests that other factors, such as a sense of burdensomeness, may also play a role in suicidality for youth involved in the legal system. This points to the need for interventions that simultaneously address multiple facets of mental health, such as self‐worth, connectedness, and future goals. These findings suggest that hope‐based interventions may be particularly beneficial for youth involved in the legal system. Hope‐focused programs, which have shown success among children and middle schoolers, could be adapted for youth with legal involvement. For example, a 5‐day school curriculum for middle schoolers demonstrated an increase in student hopefulness (Marques, Lopez, and Pais‐Ribeiro 2011). Although not targeted at adolescents, a week‐long camp for children who experienced trauma used a hope‐based curriculum and showed significantly increased hopefulness as well (Hellman and Gwinn 2017). Incorporating hope‐building activities into community re‐entry programs might strengthen resilience and improve mental health outcomes in this population. Hope‐based interventions often include goal‐setting exercises, problem‐solving strategies, and activities that promote future‐oriented thinking—all of which could help these youth counteract risk factors for suicidality and strengthen their capacity for self‐regulation.

### 4.5 Limitations

This is a cross‐sectional study, and therefore we cannot make conclusions on the mechanisms involved in these relationships. Although we account for several important potentially confounding variables, there are likely factors in each youth's background and environmental circumstances that impact their risk for substance use and suicidality that were not measured. Although the K‐CAT offers numerous benefits and efficient screening of a wide variety of mental health domains, each participant is likely administered a unique set of items. When making suicide risk formulations, responses to specific items (e.g., the presence of suicide intent and plan) are often clinically informative. The K‐CAT suicidality severity score is not directly anchored to suicidal thoughts and behaviors. For example, a score of 70 on the K‐CAT does not indicate the presence of ideation and plan without suicidal intent across participants. Rather, the K‐CAT serves as a screening tool which may be followed by more intensive, directed assessment. Furthermore, the sample size was relatively small and only included youth who had screened positively for substance use. There are limited studies of youth involved in the legal system, and despite these limitations, our findings provide insights into the role of parenting and hope on outcomes among this population.

## 5 Conclusions

Due to the cross‐sectional nature of the study, we cannot claim causality. We suggest that parental support and youth hopelessness may be important targets for prevention and intervention programs aimed at improving and addressing disparities in substance use and suicidality among youth involved in the legal system. However, we recognize the importance of future research that can directly address causality to further inform intervention targets. Hope‐based interventions have demonstrated a positive impact among children, and adapting and implementing such programs among these youth may be an important step toward improving their mental health outcomes. In addition, as substance use and suicidality disproportionately impact these youth, it is critical to further examine the mechanisms by which hope mediates the relationship between family dynamics and suicidality more deeply, potentially including additional psychological factors like perceived burdensomeness or self‐efficacy. Additionally, longitudinal studies are needed to track how changes in parental behaviors and hopefulness affect youth outcomes over time. Research could also explore how hope‐based interventions can be effectively integrated into existing juvenile justice programs, examining their potential to improve both mental health and behavioral outcomes among justice‐involved youth. By understanding and leveraging the protective role of hopefulness, researchers and clinicians can better support the unique needs of this vulnerable population.

## Conflicts of Interest

The authors declare no conflicts of interest.

### Peer Review

The peer review history for this article is available at https://www.webofscience.com/api/gateway/wos/peer-review/10.1002/mhs2.70000.

## Data Availability

Due to the legal involvement of the youth included in the study, the dataset is not made publicly available.
